# Pulmonary availability of isotretinoin in rats after inhalation of a powder aerosol

**DOI:** 10.1054/bjoc.2000.1421

**Published:** 2000-09-04

**Authors:** S M Raleigh, R D Verschoyle, C Bowskill, U Pastorino, J N Staniforth, F Steele, D Dinsdale, P Carthew, C K Lim, J Silvester, A Gescher

**Affiliations:** Medical Research Council Toxicology Unit, University of Leicester, PO Box 138, Leicester, LE1 9HN, UK; Department of Thoracic Surgery, Istituto Europeo di Oncologia, Milan, Italy; Department of Pharmacy and Pharmacology, University of Bath, Bath, UK; Cancer Research Campaign New Drug Development Office, London, UK

**Keywords:** aerosol delivery, chemoprevention, retinoids, pharmacokinetics

## Abstract

Repeated oral administration of chemopreventive retinoids such as isotretinoin over extended periods of time is associated with intolerable systemic toxicity. Here isotretinoin was formulated as a powder aerosol, and its delivery to the lungs of rats was studied with the aim to explore the possibility of minimizing adverse effects associated with its oral administration. Rats received isotretinoin orally (0.5, 1 or 10 mg kg^–1^) or by inhalation (theoretical dose ~1 or ~10 mg kg^–1^) in a nose-only inhalation chamber. Isotretinoin was quantitated by high-pressure liquid chromatography in plasma and lung tissue. The ratios of mean area of concentration-vs-time curve (AUC) values in the lungs over mean AUCs in the plasma for isotretinoin following single or repeated aerosol exposure surpassed those determined for the oral route by factors of between two (single low-dose) and five (single high-dose). Similarly, the equivalent ratios for the maximal peak concentrations in lungs and plasma obtained after aerosol exposure consistently exceeded those seen after oral administration, suggesting that lungs were exposed to higher isotretinoin concentrations after aerosol inhalation than after oral administration of similar doses. Repeated high doses of isotretinoin by inhalation resulted in moderate loss of body weight, but microscopic investigation of ten tissues including lung and oesophagus did not detect any significant aerosol-induced damage. The results suggest that administration of isotretinoin via powder aerosol inhalation is probably superior to its application via the oral route in terms of achieving efficacious drug concentrations in the lungs. © 2000 Cancer Research Campaign


*British Journal of Cancer* (2000) **83**(7), 935–940
© 2000 Cancer Research Campaign
doi: 10.1054/ bjoc.2000.1421, available online at http://www.idealibrary.com on IDEAL®

### Pulmonary availability of isotretinoin in rats after inhalation of a powder aerosol

**SM Raleigh[1], RD Verschoyle[1], C Bowskill[1], U Pastorino[2], JN Staniforth[3], F Steele[3], D Dinsdale[1], P Carthew[1], CK Lim[1], J Silvester[4] and A Gescher[1]**

[1]Medical Research Council Toxicology Unit, University of Leicester, PO Box 138, Leicester LE1 9HN UK; [2]Department of Thoracic Surgery, Istituto Europeo di Oncologia, Milan, Italy; [3]Department of Pharmacy and Pharmacology, University of Bath, Bath; UK; [4]Cancer Research Campaign New Drug Development Office, London, UK

**Summary** Repeated oral administration of chemopreventive retinoids such as isotretinoin over extended periods of time is associated with intolerable systemic toxicity. Here isotretinoin was formulated as a powder aerosol, and its delivery to the lungs of rats was studied with the aim to explore the possibility of minimizing adverse effects associated with its oral administration. Rats received isotretinoin orally (0.5, 1 or 10 mg $kg^{-1}$) or by inhalation (theoretical dose ~1 or ~10 mg $kg^{-1}$) in a nose-only inhalation chamber. Isotretinoin was quantitated by high-pressure liquid chromatography in plasma and lung tissue. The ratios of mean area of concentration-vs-time curve (AUC) values in the lungs over mean AUCs in the plasma for isotretinoin following single or repeated aerosol exposure surpassed those determined for the oral route by factors of between two (single low-dose) and five (single high-dose). Similarly, the equivalent ratios for the maximal peak concentrations in lungs and plasma obtained after aerosol exposure consistently exceeded those seen after oral administration, suggesting that lungs were exposed to higher isotretinoin concentrations after aerosol inhalation than after oral administration of similar doses. Repeated high doses of isotretinoin by inhalation resulted in moderate loss of body weight, but microscopic investigation of ten tissues including lung and oesophagus did not detect any significant aerosol-induced damage. The results suggest that administration of isotretinoin via powder aerosol inhalation is probably superior to its application via the oral route in terms of achieving efficacious drug concentrations in the lungs. © 2000 Cancer Research Campaign

**Keywords**: aerosol delivery; chemoprevention; retinoids; pharmacokinetics

Retinoids, analogues of vitamin A, are among the few substances which have been shown unequivocally in clinical trials to prevent epithelial cancers in humans (Lotan, 1996). The encouraging trial results have stimulated considerable interest in the mechanism of action, clinical pharmacology and toxicology of the retinoids. One of the most efficacious retinoids from the standpoint of chemopreventive potency is isotretinoin (13-*cis*-retinoic acid). It caused significant remissions of oral premalignant diseases (Hong et al, 1990) and reduced the occurrence of second primary tumours in patients treated previously by surgery or radiotherapy for squamous cell carcinoma of the head and neck (Hong et al, 1986). Isotretinoin has also been investigated for its potential clinical usefulness both in the prevention of lung cancer in patients with bronchial squamous metaplasia (Lee et al, 1994) and as a therapeutic agent in patients with advanced non-small cell lung cancer (Grunberg and Itri, 1987). Unfortunately the use of retinoids such as isotretinoin in the prevention or treatment of malignancies of the aerodigestive tract poses problems of tolerability. Many patients enrolled into chemoprevention trials have been unable to tolerate doses of 1 mg $kg^{-1}$ isotretinoin for periods exceeding 6 months, because of adverse dermatological effects such as dry skin, itching, skin flaking, nasal stuffiness, xerostomia and cheilitis. These reactions can be ameliorated with topical lubricants and moisturizing agents. However, in some patients these detrimental effects interfere with compliance and dose reduction is required (Warrell et al, 1995). Other manifestations of toxicity associated with retinoid treatment are headaches, hypertriglyceridaemia, formation of hyperostosis, hypercalcaemia and hepatic damage as reflected by a transient increase in serum transaminases, alkaline phosphatases and/or bilirubin. Furthermore, retinoids are potent teratogens. This toxicity spectrum necessitates pharmaceutical strategies which target retinoids to the tissue in which the malignancy is to be prevented, thus reducing systemic exposure.

Delivery of drugs to the lungs via aerosol administration has long been practised successfully in areas of therapy other than cancer, especially in the treatment of asthma. This mode of targeted delivery offers a potentially attractive approach to minimizing the systemic toxicity associated with oral or percutanous application of chemopreventive agents (Wattenberg et al, 1997). Intriguingly, it is also under investigation as a strategy to achieve a better therapeutic ratio for agents such as doxorubicin and paclitaxel in the treatment of lung cancer (Vail et al, 1999). Using the rat as experimental model we wished to test the hypothesis that delivery of isotretinoin as an aerosol is a viable strategy in its clinical development as a lung cancer chemopreventive drug. Like most retinoids, isotretinoin is water-insoluble, therefore it was formulated for this study as a powder aerosol, based on the realization that the technology pertinent to dry powder inhalation is advancing rapidly (Clark, 1995; Virchow et al, 1994). We also wanted to assess whether delivering isotretinoin via an aerosol to

*Received 27 March 2000*
*Revised 27 June 2000*
*Accepted 30 June 2000*

*Correspondence to:* A Gescher rat lungs causes unexpected adverse effects which might eventually militate against the use of this route of administration in the clinic.

### MATERIALS AND METHODS

### Isotretinoin formulation

Isotretinoin was purchased from BASF (Mannheim, Germany) and determined as > 99% pure by high-pressure liquid chromatographic (HPLC) analysis. Drug was dissolved in DMSO, cremophor, propylene glycol and water (20:8:10:62) for administration by gavage. In the inhalation study a mixture of isotretinoin and dextrose (2 or 10%) was used. The components of this mixture were carefully milled using a centrifugal mill and formulated in the absence of light. The aerosol powder was analysed using a low-angle laser light-scattering particle sizer (Malvern Mastersizer X) or an ISI Aerodynamic Particle Sizer and Diluter (ISI Incorporated, MIN). The aerosol particle size distribution was characterized by aerodynamic diameters between 1 and 5 μm with a mass median aerodynamic diameter (MMAD) of 3.4 μm. In order to determine the respirable fraction of the aerosol formulation a single-stage impinger was operated to provide a pressure drop of 4 kPa across the dry powder container. The respirable fraction, which under these conditions comprises particles with a MMAD of below approximately 6 μm, was determined as 22 ± 3% (mean ± SD of $n$ = 3).

### Animals

Female F344 rats (160 ± 10 g), supplied by Harlan UK Ltd (Bicester, UK), were maintained in negative pressure isolators and given food and water ad libitum thoughout the study. Animal husbandry was in accordance with the animal welfare guidelines stipulated by the UK Home Office and the MRC, and the project has been approved by the Leicester University Ethical Review and Animal Welfare Committee.

### Inhalation exposure

Groups of four rats were exposed to the isotretinoin powder aerosol under subdued lighting for periods of 5, 10, 20 or 40 min in a nose-only inhalation chamber. Animals were placed in restraining cones in the inhalation chamber, which was tightly linked to a specially engineered perspex elutriator. Powder aerosol was pushed into the bottom of the elutriator through a side arm, to which a powder-filled decapitated 50 ml plastic syringe was attached. The movement of the plunger was controlled by an automatic timing device linked to a syringe pump (Razel Scientific Instruments Inc, Stanford, CA, USA). Delivery of powder aerosol into the elutriator was pulsed for 20-s periods every 2.5 min during the entire exposure period. The powder was distributed by air, pumped at a flow rate of approximately 10 l $min^{-1}$, into the elutriator through three entry points at its base. The particle load in the air reaching the rats was measured gravimetrically for the duration of exposure with a collection filter assembly containing a Millipore filter placed in an empty nose cone, through which air was drawn at 100, 250 or 1000 ml $min^{-1}$. In several experiments the isotretinoin content in the mixture was checked by HPLC analysis. The concentration of isotretinoin in the air was 0.26 ± 0.12 mg $l^{-1}$ of air in 20 exposures during the low-dose inhalation experiments using the 2% isotretinoin formulation, and 2.4 ± 0.8 mg $l^{-1}$ (mean ± SD) of air in 30 exposures during the high-dose inhalation experiments using the 10% isotretinoin formulation. Animals were subjected to either a single exposure to the aerosol powder or repeated exposure for 5–10 days. Care was taken to remove aerosol powder from the animals' fur by exposing them to a directed stream of air for 30 s after their removal from the inhalation chamber. Nevertheless, some powder aerosol remained visibly in the fur surrounding the animals' head and was thus available for oral ingestion by the animals when they groomed themselves post-exposure. Therefore isotretinoin levels after inhalation reflect a composite of inhaled and some orally ingested compound. It is likely that the oral component of this composite affects isotretinoin levels in the plasma more prominently than those in the lungs. In some experiments animals remained in the restraining cages up to the time of killing in order to minimize oral ingestion. The dose administered via inhalation was estimated from the aerosol concentration, amount of isotretinoin per litre of air and respiratory minute volume (RMV) for the rat (Guyton, 1947), by the following formula:

$$\text{dose} = \text{aerosol concentration} \times \text{RMV} \times \text{exposure time} \times \text{body weight}^{-1}$$

The assumption of this approach is that the inspired aerosol is deposited completely in the lungs. Therefore, the doses referred to in the following in the context of the inhalation experiments have to be viewed as 'theoretical' doses.

### Oral dosing

Groups of at least four rats received isotretinoin by gastric lavage once or repeatedly for 14 days. The oral doses were 0.5, 1 and 10 mg $kg^{-1}$.

### Sample preparation, HPLC analysis and measurement of the area under the drug concentration-vs-time curve (AUC)

In the oral administration study animals were killed at 0.5, 1, 6 and 24 h after gavage, in some experiments 2, 4 and 48 h points were included. In the inhalation study rats were killed at 0.25, 0.5, 1, 2 and 4 h after completion of the final exposure. Blood was obtained by cardiac puncture under terminal anaesthesia, collected into heparinized tubes and centrifuged to yield plasma. Lung tissue was excised and weighed, and plasma and tissue were stored at –80°C until analysis. Retinoids were extracted from tissue and plasma into an equal volume of hexane and quantitated by a normal phase HPLC method with UV detection as described by Lanvers et al (1996) using arotenoid ethylsulphone as internal standard. The internal standard and reference material for the isotretinoin metabolite-4-oxo-isotretinoin were kindly provided by M Klaus (Hoffmann-La Roche, Basel, Switzerland). A destructive sampling scheme was employed. The mean areas under the plasma or lung concentration vs time curves (AUC) between 0 and 24 h in the case of the oral administration, or from 0–4 or 6 h for the inhalation exposure study were computed from the mean plasma and tissue levels by the trapezoidal rule (Rowland and Tozer, 1995). Standard errors for the AUC values were calculated from the variances of drug/metabolite levels at individual time points

© 2000 Cancer Research Campaign and the number of animals used ($n$) per time point, by the following formula (Gagnon and Peterson, 1998):

$$\text{Variance (AUC)} = \Sigma\,[(\text{trapezoidal weights}^2) \times (\text{variance of values at each time-point} \times n^{-1})]$$

Values of peak levels and doses quoted in the Results represent the mean ± SD.

### Toxicology

Groups of four rats each were exposed to either milled dextrose powder, the excipient in the isotretinoin aerosol formulation, or to the isotretinoin powder, repeatedly for 5 days (high-dose) or 10 days (low-dose). Each exposure period was 5 min, and animal weights were recorded daily. The theoretical doses were approximately 10 mg kg$^{-1}$ isotretinoin with 90 mg kg$^{-1}$ dextrose in the high-dose experiments (10% formulation) and 2 mg kg$^{-1}$ isotretinoin with 120 mg kg$^{-1}$ dextrose in the low-dose experiments (2% formulation). In control experiments equivalent doses of dextrose only were inhaled. Animals were killed 1, 3 or 7 days after completion of the final inhalation exposure. Lung, liver, kidney, thymus, pancreas, spleen, heart, skin, oesophagus and stomach tissues were removed and fixed in buffered formalin and stained with haematoxylin and eosin for histopathological examination by light microscopy. Concentrations of alkaline phosphatase (ALP) in the plasma were determined using an ALP Optimized Kit (Sigma Chemical Company, Poole, UK).

### RESULTS

### Lung levels of isotretinoin after powder aerosol administration

The suitability of isotretinoin formulated with dextrose as a powder aerosol for inhalation was tested in rats which inhaled isotretinoin at either low or high theoretical doses, which were approximately 1 mg kg$^{-1}$ and 10 mg kg$^{-1}$, respectively. Figure 1 shows that inhalation of the aerosol furnished measurable isotretinoin levels in the lung. Isotretinoin was also found in the plasma. Isomerization of the drug to all-*trans*- or 9-*cis*-retinoic acids was not observed either in the lungs or the plasma (not shown), but in the high-dose exposure experiments, the isotretinoin metabolite 4-oxo-isotretinoin was found. The $AUC_{(0–4\ h)}$ values for isotretinoin in the lungs increased linearly with inhalation dose after single exposure (Figure 2), which indicates that the administered dose increased proportionally with exposure time. Even though the linearity of this relationship was less obvious after repeated dosing (Figure 2), these results suggest broadly linear pharmacokinetics for inhaled isotretinoin at the dose-range administered in this study, supporting the potential feasibility of this approach.

### Comparison of aerosol and oral administration of isotretinoin

Animals also received isotretinoin by gavage at low (0.5 mg kg$^{-1}$ single, 1 mg kg$^{-1}$ repeated) and high (10 mg kg$^{-1}$ single or repeated) doses in order to allow comparison of lung and plasma levels after inhalation exposure with those seen after the conventional mode of isotretinoin administration. The lung concentration-vs-time curves

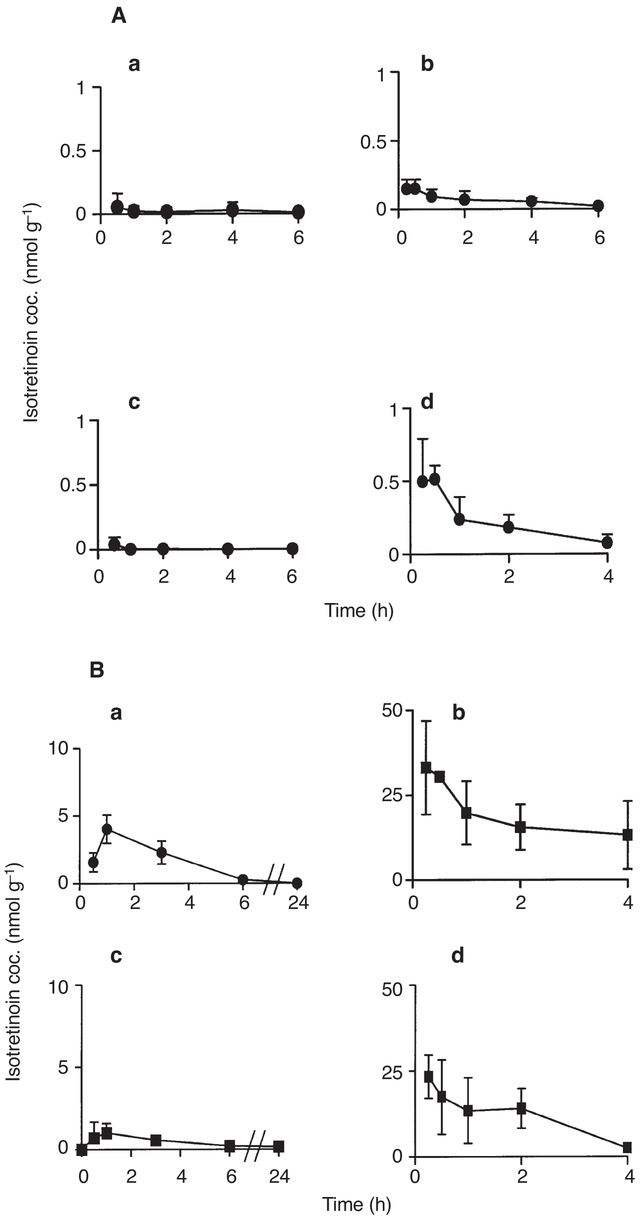

**Figure 1** Isotretinoin levels in rat lung after administration of isotretinoin either at a low (**A**) or high dose (**B**), given orally (a, c) as a single dose (a) or daily for 14 days (c), or after termination of exposure to isotretinoin powder aerosol (b, d) given once (b) or repeatedly (d), daily for 10 (**A**) or 7 days (**B**). The doses were as follows: (**A**) oral dose 0.5 once or 1 mg kg$^{-1}$ repeatedly; inhalation exposure to the 2% isotretinoin powder formulation for 5 min once or repeatedly (theoretical dose approximately 1 mg kg$^{-1}$); (**B**) oral dose 10 mg kg$^{-1}$ once or repeatedly, inhalation exposure to the 10% isotretinoin powder formulation for 5 min once or repeatedly (theoretical dose approximately 10 mg kg$^{-1}$). Values are the mean ± SD of four animals in Figure 1 indicate higher concentrations of isotretinoin in the lungs after aerosol administration at both low (Figure 1A) and high doses (Figure 1B) compared to those achieved after oral administration of the drug at comparable doses.

Comparison of the corresponding AUC and $C_{max}$ values in lung tissue and plasma suggests that drug availability was higher in the lungs following aerosol administration at both dose-levels (see Table 1). Figure 3 shows the ratios of mean lung AUC or $C_{max}$

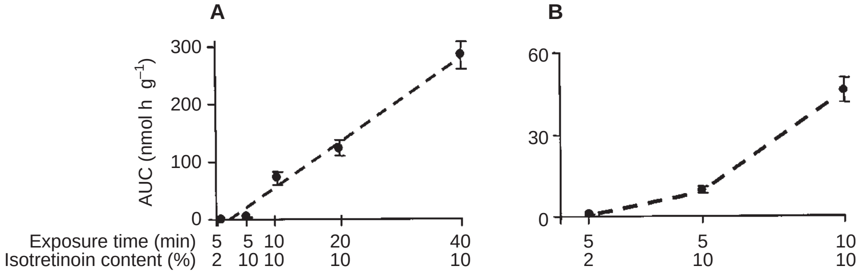

**Figure 2** Relationship between isotretinoin inhalation dose and isotretinoin lung AUC values after single (**A**) or repeat exposure (**B**) of rats to the isotretinoin powder aerosol. Lung $AUC_{(0-4\ h)}$ values were calculated from lung-concentration-vs-time curves with five time-points after termination of exposure. Numbers of multiple daily exposures in (**B**) were 10 for the lowest dose, five for the intermediate and seven for the highest daily dose. Error bars for the AUC values were derived from the variances of the individual time points. The individual isotretinoin lung level data points in the lung-concentration-vs-time curves were characterized by SDs varying between 18 and 55% of the mean. Regression analysis of the line in (A) furnishes a coefficient of 0.9879

| Dose | Route | $AUC_{plasma}$ (nmol h $ml^{-1}$) | $AUC_{lung}$ (nmol h $g^{-1}$) | $C_{max,\ plasma}$ (nmol $ml^{-1}$) | $C_{max,\ lung}$ (nmol $g^{-1}$) |
|---|---|---|---|---|---|
| Low single | p.o. | 0.96 ± 0.006 | 0.14 ± 0.10 | 0.38 ± 0.05 | 0.06 ± 0.05 |
| | aerosol | 1.44 ± 0.13 | 0.39 ± 0.05 | 0.36 ± 0.08 | 0.15 ± 0.07 |
| High single | p.o. | 35.0 ± 3.2 | 12 ± 2 | 12.1 ± 1.0 | 4.0 ± 1.1 |
| | aerosol | 40 ± 6 | 72 ± 12 | 14.0 ± 9.0 | 33 ± 14 |
| Low repeated | p.o. | 0.73 ± 0.06 | 0.03 ± 0.02 | 0.23 ± 0.11 | 0.04 ± 0.03 |
| | aerosol | 2.10 ± 0.47 | 0.84 ± 0.15 | 0.67 ± 0.85 | 0.51 ± 0.10 |
| High repeated | p.o. | 15.1 ± 1.7 | 3.3 ± 0.7 | 5.7 ± 3.7 | 0.7 ± 0.6 |
| | aerosol | 50.1 ± 7.8 | 45.9 ± 4.6 | 22.1 ± 17.0 | 23.3 ± 6.3 |

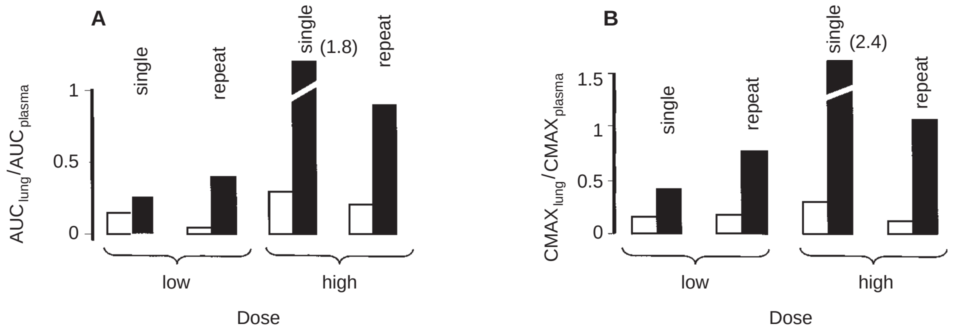

**Figure 3** Ratios of mean $AUC_{(0-4\ h)}$ values of isotretinoin in the lung ($AUC_{lung}$) over mean $AUC_{(0-24\ h)}$ values in the plasma ($AUC_{plasma}$) (**A**), and ratios of mean peak concentrations of isotretinoin in the lung ($C_{max,\ lung}$) over those in the plasma ($C_{max,\ plasma}$) (**B**), in rats after administration of isotretinoin p.o. (open bars) or by inhalation of powder aerosol (closed bars). Isotretinoin was given at a low or high dose once or repeatedly. Table 1 shows the AUC and $C_{max}$ values used for the ratio calculation. The doses were as follows: (**A**) oral dose 0.5 once and 1 mg $kg^{-1}$ daily for 14 days; inhalation exposure to the 2% isotretinoin powder formulation for 5 min once or daily for 10 days (theoretical dose approximately 1 mg $kg^{-1}$); (**B**) oral dose 10 mg $kg^{-1}$ once or daily for 14 days, and inhalation exposure to the 10% isotretinoin powder formulation for 5 min once or daily for 7 days (theoretical dose approximately 10 mg $kg^{-1}$)

values of drug over mean plasma AUC or $C_{max}$ values, measured after single or repeated administration by either the oral or inhalation routes. The ratios of the AUCs in the aerosol exposures exceed those computed for the oral route by factors of between two in the case of the single low-dose to five for the single high-dose. Similarly the ratios for the $C_{max}$ values obtained in the aerosol exposures surpass those seen after p.o. administration by factors of between two and eight.

In additional experiments, rats underwent single exposure to isotretinoin powder aerosol at a range of higher-dose levels up to approximately 40 mg $kg^{-1}$, and the resultant ratios of mean lung isotretinoin AUC or $C_{max}$ values over mean plasma isotretinoin AUC or $C_{max}$ values were consistently 3- to 8-fold higher than those computed for the high oral dose (results not shown). Surprisingly, plasma isotretinoin levels after aerosol administration were similar to, or even above, those after oral dosing. This finding is most likely a corollary of the fact that plasma levels after inhalation reflect a composite of inhaled and ingested isotretinoin. As described in Methods, when the rats groomed themselves post-exposure, they ingested isotretinoin powder which had inevitably been lodged in the fur around the neck during the inhalation period. Therefore, plasma levels post-aerosol delivery in the rat are likely to overestimate systemic bioavailibility of the isotretinoin aerosol as an intrinsic consequence of the experimental design.

Comparison of the levels and AUC values depicted in Figures 1 and 3 intimates that concentrations achieved in the lungs after repeated high doses of isotretinoin were lower than those obtained after single administration, irrespective of route of administration. This difference suggests that prolonged exposure to high doses of isotretinoin does not lead to accumulation of the drug in rat lung. Levels of the isotretinoin metabolite 4-oxo-isotretinoin were

*© 2000 Cancer Research Campaign*

below the limit of quantitation after the single oral high dose of isotretinoin, whereas after multiple oral dosing the lung AUC of 4-oxo-isotretinoin was 5.1 ± 0.4 nmol h $g^{-1}$ tissue (mean ± SD of *n* = 3). For the single inhalation exposure at the high dose, the lung AUC for 4-oxo-isotretinoin was 4.5 ± 1.2 nmol h $g^{-1}$, the corresponding value after multiple exposure was 7.4 ± 1.5 nmol h $g^{-1}$, constituting an increase of 64% over single dosing. A similar elevation was seen in plasma 4-oxo-isotretinoin levels. Thus the 4-oxo-isotretinoin AUC values inversely mirror the changes in parent drug AUC described above, in that they were considerably higher after repeated than after single doses of isotretinoin. These data are consistent with the notion that – irrespective of route of administration – prolonged exposure to high concentrations of isotretinoin, at least in the rat, induces its own metabolic disposal.

### Safety of isotretinoin powder aerosol

We wished to assess, in a preliminary fashion, the safety of the isotretinoin powder aerosol in order to allow judgement as to the feasibilty of its potential development for clinical trial. To that end animals were exposed to the aerosol powder once or daily for either 5 or 10 consecutive days. Control animals inhaled dextrose, the excipient used in the aerosol formulation. Lung, liver, kidney, thymus, pancreas, spleen, heart, skin, oesophagus and stomach tissues did not show any signs of aerosol-induced morphological alterations. Animals which received the high dose of isotretinoin aerosol (10% isotretinoin formulation for 5 min) daily for 5 days started to lose weight after the fourth dose, with the weight nadir occurring on day 7, two days after termination of exposure, by which time they had lost a mean of 12% of their body weight. Subsequently, they commenced to gain weight again. Control rats exposed to dextrose powder only, animals exposed to isotretinoin powder aerosol at the low dose (2% isotretinoin formulation for 5 min daily) for 10 days, or animals which received 10 mg $kg^{-1}$ isotretinoin p.o. for 14 days did not lose weight. Plasma ALP activity was measured immediately after the final exposure of rats to 2% isotretinoin aerosol for 5 min periods on 10 consecutive days. ALP concentrations in units $L^{-1}$ were 201 ± 8 (*n* = 8) in control rats, 202 ± 5 (*n* = 13) in dextrose-exposed animals and 275 ± 6 (*n* = 29) in rats exposed to isotretinoin, which constitutes a modest but significant increase of 36% compared to controls ($P <$ 0.001). Elevation of ALP activity in the plasma has been shown in the original toxicity study of isotretinoin in rats at doses of 4 mg $kg^{-1}$ or more given p.o. daily for 4 weeks, and this elevation has been tentatively associated with the effects of excess vitamin A on bone (Hixson et al, 1979).

### DISCUSSION

The work outlined above describes, for the first time, the use of a powder aerosol formulation as a delivery system for a lung cancer chemopreventive agent. The pharmaceutical arguments in favour of designing a powder aerosol to target drugs to the lungs are compelling. They include the physical and chemical stability of the active ingredient in a powder formulation, uniformity and reproducibility of the inhaled dose and deep-lung penetration of the inhaled small drug particles. These considerations are especially pertinent for agents such as isotretinoin, which is highly water-insoluble and exquisitely susceptible to light-induced decomposition when dissolved. The results described in this paper show clearly that the ratios of drug concentrations in the lungs over those in the plasma were invariably higher after inhalation than after oral administration. This difference was observed irrespective of the magnitude of dose administered or of whether ratio computation was based on AUC or peak concentration values. Furthermore, repeated aerosol administration was not confounded by drug accumulation in the lungs. Thus the study supports the hypothesis that for the chemoprevention of malignancies of the upper aerodigestive tract inhalation of isotretinoin powder aerosol is preferable to its oral administration, because the aerosol yields a superior pharmacokinetic profile.

It is important to consider how the isotretinoin concentrations observed in this study in rats compare with those achievable in humans. A single oral dose of 0.5 mg $kg^{-1}$ isotretinoin in humans resulted in a mean peak plasma concentration of 0.83 nmol $ml^{-1}$ (Kerr et al, 1982). The plasma levels described here in rats, 1.5 nmol $ml^{-1}$ and 0.4 nmol $ml^{-1}$ after low oral or aerosol inhalation doses, respectively, are of the same order of magnitude. The ratios of lung to plasma drug levels observed here in rats may well be representative of other species and not restricted to this animal model. For example, published isotretinoin peak levels in the lungs and plasma of mice after isotretinoin 10 mg $kg^{-1}$ p.o. (Kalin et al, 1982) permit calculation of a ratio of mean peak lung to plasma concentrations of 0.24, compared to 0.34 described here for the same oral dose in the rat. Applying this ratio to a speculative computation of peak lung levels in humans after the 0.5 mg $kg^{-1}$ oral dose of isotretinoin yields approximately 0.20 nmol isotretinoin per gram of lung. If inhalation of isotretinoin in humans leads to lung levels similar to those seen in our rats after repeated inhalation administration of the low dose, one could expect human peak lung levels at least 2.5-fold higher than those seen after oral medication. This value is probably a considerable underestimate, as exposure of rats to a therapeutic aerosol in an inhalation chamber is undoubtedly an imperfect model for human aerosol administration. Nevertheless, concentrations of isotretinoin of this order of magnitude achieved repeatedly in human lungs may be sufficient to prevent cancer. Most of the effects of retinoids are mediated via the retinoic acid receptors (RAR) and retinoid X receptors (RXR), nuclear steroid hormone receptors which act as transcription factors. Upregulation of one specific receptor type, RAR-β, has been correlated with the chemopreventive efficacy of retinoids (Ahn et al, 1995; Lotan et al, 1995). In experiments in human bronchial epithelial cells in vitro upregulation of RAR-β protein expression was elicited by 2 nmol $ml^{-1}$ retinoic acid within a 12 h period (Boyle et al, 1999). The present study indicates that concentrations of this order of magnitude might be achievable in human lung tissue after inhalation of isotretinoin. Ultimately, the contention that chemopreventive levels of isotretinoin can be achieved in the human lung after aerosol delivery remains to be investigated in the clinic.

The observations made here as to the feasibility of delivery of chemopreventive agents via powder aerosol complement the conclusions drawn from two recent preclinical studies in which the glucocorticoid budesonide and the isotretinoin isomer all-trans retinoic acid (ATRA) have been administered as liquid aerosols via the inhalation route. Exposure of mice to aerosolized budesonide significantly inhibited formation of benzo(*a*)pyrene-induced pulmonary adenomas (Wattenberg et al, 1997), and inhalation by mice of ATRA liposomes induced both the concentration and activity of transglutaminase in alveolar macrophages (Parthasarathy et al, 1999). The latter study differs from ours in that

© 2000 Cancer Research Campaign 

administration of ATRA as liquid liposomal aerosol furnished detectable drug levels only in the lungs, but not in the plasma. In contrast, isotretinoin powder aerosol administration led to measurable retinoid levels also in the plasma. This discrepancy could be related to differences in absorption from the gastrointestinal tract of retinoid ingested orally during aerosol exposure. It is conceivable that absorption of liposomally entrapped ATRA in the study by Parthasarathy et al (1999) was much more limited than that of powdered isotretinoin. We believe that the advantageous pharmaceutical properties of a dry powder formulation as compared to liposomes suspended in liquid with potentially complicated stability and drug-release characteristics should be considered carefully when the retinoid aerosol formulations are eventually evaluated for advancement into clinical development.

Animals which inhaled isotretinoin aerosol powder repeatedly at the high dose suffered moderate weight loss. In contrast, repeated administration of a comparable oral dose did not elicit this effect. The basis of the drug-induced weight loss after inhalation exposure of high-dose isotretinoin could not be established, as it was not matched by significant alterations in the morphology of a variety of organs which were investigated. Importantly, our studies render it unlikely that the lungs are the source of this adverse effect. Nevertheless, the possibility of unexpected adverse effects specific to lung delivery has to be borne in mind when future clinical trials of isotretinoin powder aerosol are planned.

Targeting agents to the desired site of action is a priority area in the portfolio of strategies currently being pursued in the optimization of the development of cancer chemopreventive drugs. The work outlined here in concert with related recent studies (Wattenberg et al, 1997; Parthasarathy et al, 1999) suggests that specific delivery of agents to the lungs for chemoprevention of lung cancer is an attractive and exciting option, at least for glucocorticoids and retinoids.

### ACKNOWLEDGEMENTS

This work was supported by a grant from the Cancer Research Campaign and a travel bursary (to SMR) from the EORTC Pharmacology and Molecular Mechanisms Group. We thank J Boos and C Lanvers (University of Muenster, Germany) for help with the development of the assay of retinoids in lung tissue, M Festing (MRC Toxicology Unit) for advice concerning the statistical evaluation and M Williams for useful discussions. The work constitutes a collaboration under the auspices of the EORTC Pharmacology and Molecular Mechanisms Group.

### REFERENCES

Ahn MJ, Langenfeld J, Moasser MM, Rusch V and Dmitrovsky E (1995) Growth suppression of transformed human bronchial epithelial cells by all-*trans*-retinoic acid occurs through specific retinoid receptors. *Oncogene* **11**: 2357–2364

Boyle JO, Langenfeld J, Lonardo F, Sekula D, Reczek P, Rusch V, Dawson MI and Dmitrovsky E (1999) Cyclin D1 proteolysis: a retinoid chemoprevention signal in normal, immortalized, and transformed human bronchial epithelial cells. *J Natl Cancer Inst* **91**: 373–379

Clark AR (1995) Medical aerosol inhalers – past, present and future. *Aerosol Sci Tech* **22**: 374–391

Gagnon RC and Peterson JJ (1998) Estimation of confidence intervals for area under the curve from destructively obtained pharmacokinetic data. *J Pharmacokinet Biopharm* **26**: 87–102

Guyton AC (1947) Measurement of the respiratory volumes of laboratory animals. *Am J Physiol* **150**: 70–77

Grunberg S and Itri L (1987) Phase II study of isotretinoin in the treatment of advanced non small cell lung cancer. *Cancer Treat Rep* **71**: 1097–1098.

Hixson EA, Burdeshaw JA, Denine EP and Harrison SD (1997) Comparative subchronic toxicity of all-*trans*- and 13-*cis*-retinoic acid in Sprague-Dawley rats. *Toxicol Appl Pharmacol* **47**: 359–365

Hong WK, Endicott J, Itri LM, Doos W, Batsakis JG, Bell R, Fofonoff S, Byers RM, Atkinson EN, Vaughan C, Toth BB, Kramer A, Dimery IW, Skipper P and Strong S (1986) 13-*Cis* retinoic acid in the treatment of oral leukoplakia. *N Engl J Med* **315**: 1501–1505

Hong WK, Lippman SM, Itri LM, Karp DD, Lee JS, Byers RM, Schantz SP, Kramer AM, Lotan R, Peters LJ, Dimery IW, Brown BW and Goepfert H (1990) Prevention of second primary tumors with isotretinoin in squamous-cell carcinoma of the head and neck. *N Engl J Med* **323**: 795–801

Kalin JR, Wells MJ and Hill DL (1982) Disposition of 13-cis retinoic acid and N-(2-hydroxyethyl)retinamide in mice after oral dosing. *Drug Metab Dispos* **10**: 391–398.

Kerr IG, Lippman ME, Jenkins J and Myers CE (1982) Pharmacology of 13-cis-retinoic acid in humans. *Cancer Res* **42**: 2069–2073

Lanvers C, Hempel G, Blaschke G and Boos J (1996) Simultaneous determination of all-trans-, 13-cis-, and 9-cis-retinoic acid, their 4-oxo metabolites and all-trans-retinol in human plasma by high-performance liquid chromatography. *J Chromatogr* **685**: 233–240

Lee JS, Lippman SM, Benner SE, Lee JJ, Ro JY, Lukeman JM, Morice RC, Peters EJ, Pang AC, Fritsche HA and Hong WK (1994) Randomized placebo-controlled trial of isotretinoin in chemoprevention of bronchial squamous metaplasia. *J Clin Oncol* **12**: 937–945

Lotan R (1996) Retinoids in cancer chemoprevention. *FASEB J* **10**: 1031–1039.

Lotan R, Xu XC, Lippman SM, Ro JY, Lee JS, Lee JJ and Hong WK (1995) Suppression of retinoic acid receptor-beta in premalignant oral lesions and its up-regulation by isotretinoin. *N Engl J Med* **332**: 1405–1410

Parthasarathy R, Gilbert B and Mehta K (1999) Aerosol delivery of liposomal all-trans-retinoic acid to the lungs. *Cancer Chemother Pharmacol* **43**: 277–283

Rowland M and Tozer TN (1995) *Clinical Pharmacokinetics – Concepts and Applications*, pp 465–472. Williams and Wilkins: Media, Pennysylvania

Vail DM, Hershey AE, Kurzmann I, Bohling C, Forrest LJ, Stonerook M, Placke ME and Imondi AR (1999) Inhalation chemotherapy for macroscopic primary or metastatic lung tumors: proof of principle. *Proc Am Assoc Cancer Res* **40**: 416.

Virchow JC, Kroegel C and Matthys H (1994) Antiasthma drug delivery – what is on the horizon? *Clin Pharmacokinet* **27**: 85–93.

Warrell RP, Pastorino U and Decensi A (1995) Clinical toxicology of the retinoids. In *Retinoids in Oncology, ESO Monograph*, Degos L and Parkinson DR (eds), pp 67–71. Springer Verlag: Berlin, Heidelberg, New York

Wattenberg LW, Wiedmann TS, Estensen RD, Zimmerman CL, Steele VE and Kelloff GJ (1997) Chemoprevention of pulmonary carcinogenesis by aerosolized budesonide in female A/J mice. *Cancer Res* **57**: 5489–5492.

 © 2000 Cancer Research Campaign
